# Electron beam-induced athermal nanowelding of crossing SiO_*x*_ amorphous nanowires†

**DOI:** 10.1039/d1ra08176d

**Published:** 2022-02-21

**Authors:** Yuchen Zheng, Liang Cheng, Jiangbin Su, Chuncai Chen, Xianfang Zhu, Hang Li

**Affiliations:** Department of Physics, Xiamen University Xiamen 361005 People's Republic of China zhux@xmu.edu.cn; China–Australia Joint Laboratory for Functional Nanomaterials, Xiamen University Xiamen 361005 People's Republic of China; Experiment Center of Electronic Science and Technology, School of Microelectronics Science and Control Engineering, Changzhou University Changzhou 213164 People's Republic of China; Department of Physics, College of Civil Engineering, MinNan University of Science and Technology Shishi 362700 People's Republic of China; State Key Lab of Physical Chemistry of Solid Surfaces, Collaborative Innovation Centre of Chemistry for Energy Materials, State-Province Joint Engineering Laboratory of Power Source Technology for New Energy Vehicle, Engineering Research Center of Electrochemical Technology, Ministry of Education, College of Chemistry and Chemical Engineering, Xiamen University Xiamen 361005 People's Republic of China

## Abstract

Nanowelding of two crossing amorphous SiO_*x*_ nanowires induced by uniform electron beam irradiation at room temperature was demonstrated in an *in situ* transmission electron microscope. It was observed that, under the electron beam irradiation, the amorphous nanowires became unstable driven by nanocurvature non-uniformly distributed over the nanowire surface centered around the crossing site of the nanowires. Such an instability of the nanowires could give rise to an athermal fast and massive migration of atoms nearby the surface centered around the crossing site, and thus the two crossing nanowires become gradually welded. The existing knock-on mechanism and molecular dynamics simulations seem inadequate to explain the observed athermal migration of the surface atoms and the resulting structural change at the nanoscale. To elucidate the observed phenomena of nanowelding, a mechanism of athermal atomic diffusion driven by the effects of the nanocurvature as well as the athermal activation of the electron beam was proposed and simulated. The simulation revealed the detailed process of the nanowelding and corresponding effects of the nanocurvature and athermal activation of the electron beam. In doing so, the nanowelding parameters became predictable, controllable, and tunable to a desired welding effect.

## Abstract 2

Athermal welding of crossing SiO_*x*_ nanowires under e-beam irradiation is *in situ* observed by TEM. A relevant simulation considering nanocurvature effect and athermal activation effect gives the corresponding 3D structural evolution and the velocity field of atom migration.

## Graphical abstract

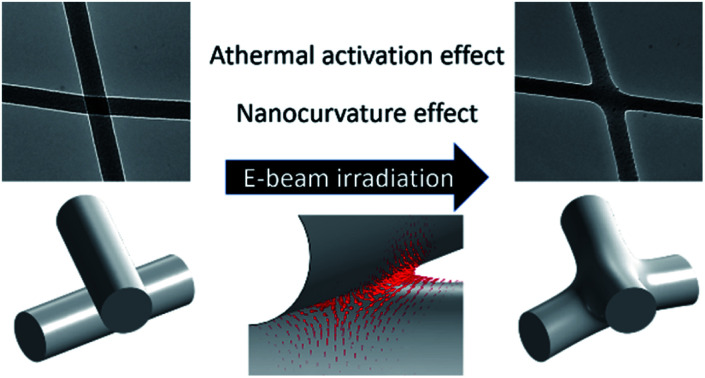

## Introduction

In recent years, low dimensional nanostructures (LDNs) have attracted considerable attention due to their numerous potential applications in nanodevices and nanotechnology.^1–4^ Nevertheless, due to uncontrollability in their growth and fabrication, the as-grown or as-fabricated LDNs normally do not possess desired shapes, structures or properties. Therefore, a followed-up nanoprocessing, for instance, nanowelding of LDNs,^5^ becomes crucial to their applications. In the existing studies, the nanowelding techniques have been successfully applied to a variety of materials. Metal nanostructures^6–10^ were welded together through mechanical contact,^6^ semiconductor nanostructures^11^ were welded under a temperature below the melting point, and ceramic nanostructures^12^ were welded through electron irradiation. The other nanowelding methods found include sintering,^7^ ultrasonic welding,^13^ resistance welding,^8^ light welding,^9^ and laser beam.^10^ These methods are normally designed to make thousands of joints simultaneously in the case of heating, but are hardly controllable to weld or join two nanostructures just at one specifically targeted location at room temperature, while leaving the other parts intact. Thus, electron beam (e-beam) welding becomes an attractive solution because it can be used at the intended point without substantially affecting the surrounding materials.

*In situ* energetic e-beam in the transmission electron microscopy (TEM) is a unique tool that can be used not only for characterizing the structure and morphology of LDNs at a resolution down to atomic distance but also for beam-induced structure change (nanoinstability or nanoprocessing).^14,15^ Using this technique, Xu *et al.*^14^ successfully welded Au nanowires (NWs) by irradiating the point, at which two single-crystalline Au NWs were crossed with a high-intensity e-beam. Yang *et al.*^16^ revealed that a mild e-beam irradiation could induce controllable welding of local nanospots in carbon nanotubes. Terrones *et al.*^17^ showed that several types of junctions between two carbon nanotubes (CNTs) can be formed by welding at 800 °C with the help of an e-beam irradiation. Up till now, the e-beam welding technique has been mainly focused on crystalline metallic and semiconductor NWs or CNTs.^14,16–19^ However, there have been few experiments found so far to deal with the welding of amorphous LDNs under the e-beam irradiation, and thus the underlying physical mechanism for all the nanowelding has not been explored systematically or adequately yet. In the existent attempts, due to the fact that there is no such a theory to account for the non-equilibrium, amorphous, and non-linear nature of LDNs, particularly under energetic beam irradiation, people normally have to resort to the current theories such as classical knock-on mechanism^20^ and some related molecular dynamics simulations^14,15,21,22^ to explain and predict the dynamic atomic defect creation and annihilation and atom transport processes as beam-induced in LDNs.^14,15,20–22^ However, the current theories cannot offer an explanation for all the observed, beam-induced nanophenomena, particularly for these such as the plastic flow or wetting of amorphous SiO_*x*_ (a-SiO_*x*_) NWs^23^ and carbon nanotubes^24^ and the accelerated shrinkage of nanocavities in silicon along with its cavity-near-surface preferential amorphization.^25,26^ This is because the current theories are established at the first place on the consideration of the nature of equilibrium, symmetry, periodicity, and linearity of the bulk crystalline structure or its approximation whereas the beam (including electron, ion and photon beams) induced nanophenomena are intrinsically of non-equilibrium, amorphous, and non-linear nature. For the above reasons, we proposed novel concepts including the nanocurvature effect^26,27^ and the beam-induced athermal activation effect^26,28^ which can well explain the above observed beam-induced nanophenomena and nanoprocessings of LDNs.^23–26,29^ Furthermore from the concepts, we also predicted the beam-induced near-surface nanowelding of two crossing amorphous NWs driven by non-uniform nanocurvature over the surface nearby their contacting point. Nevertheless, so far, there has been no experimental work reported to confirm the above prediction.

From the above considerations, in this study, we reported our experimental findings on the nanowelding of two crossing a-SiO_*x*_ NWs during *in situ* uniform e-beam irradiation in TEM at room temperature. The experiment confirmed that under the e-beam irradiation, the a-SiO_*x*_ NWs became indeed unstable driven by non-uniform nanocurvatures over the surface nearby their contacting point. Such an instability could induce an amazing athermal migration of atoms nearby the NW surface whereby the two crossing NWs were gradually welded. The classical knock-on mechanism and related molecular dynamics simulations were found to be inadequate to account for the observed athermal surface migration of atoms and the resulting structural change at the nanoscale. To explain the observed nanowelding phenomenon, we herein proposed a mechanism of athermal atomic diffusion driven by the nanocurvature effect and the beam-induced athermal activation effect. Furthermore, a computer simulation was conducted. The simulation revealed the detailed process of the nanowelding and the corresponding effects of the nanocurvature and athermal activation of the e-beam.

## Experimental

The straight a-SiO_*x*_ NWs with smooth surface were synthesized by our improved chemical vapor deposition set-up, where *x* was determined to be 2.3.^30^ Then, they were dispersed in 99.7% ethanol for 30 min, and finally collected on electron-transparent, holey carbon film (with large holes) on Cu grids for TEM studies. The as-prepared specimens were then irradiated at room temperature, and the structure evolutions were *in situ* observed in a field emission TEM (FEI Tecnai F-30) operating at 300 kV. The beam was kept perpendicular to the axes of the wires using the practice, as reported in ref. 31. It was estimated that the penetration depth^32^ of such electrons exceeded the thickness of TEM specimens (*i.e.*, the two crossing NW diameter in this case). The two crossing, straight and clean NW segments were ideal candidates for the investigation of nanowelding due to a non-uniform distribution of nanocurvature over the surface nearby their contacting point. During the irradiation, the current density at the specimen was kept to approximately 1 A cm^−2^ (flux: 6.25 × 10^4^ nm^−2^ s^−1^) which was uniform over an area larger than the zone observed. Such an e-beam of low-intensity^33^ appeared to enhance the diffusion of atoms along the surface of a-SiO_*x*_ NWs, where the evaporation of atoms can be negligible. For the TEM observation, the beam intensity was periodically spread to an around 100 times weaker intensity for the observation or taking a picture. In this way, the irradiation effect during the observation or taking a picture can be minimized to a negligible degree and at the same time the image contrast can also be improved. Also note that during the electron irradiation, the beam was expected to heat the specimen by no more than a few degrees^20,23,33^ due to their high ratio of surface to volume, and the dominant irradiation effect should be non-thermal. Therefore, we considered that the irradiated NWs essentially remained at room temperature throughout the irradiation.

## Results and discussion

**Fig. 1:**
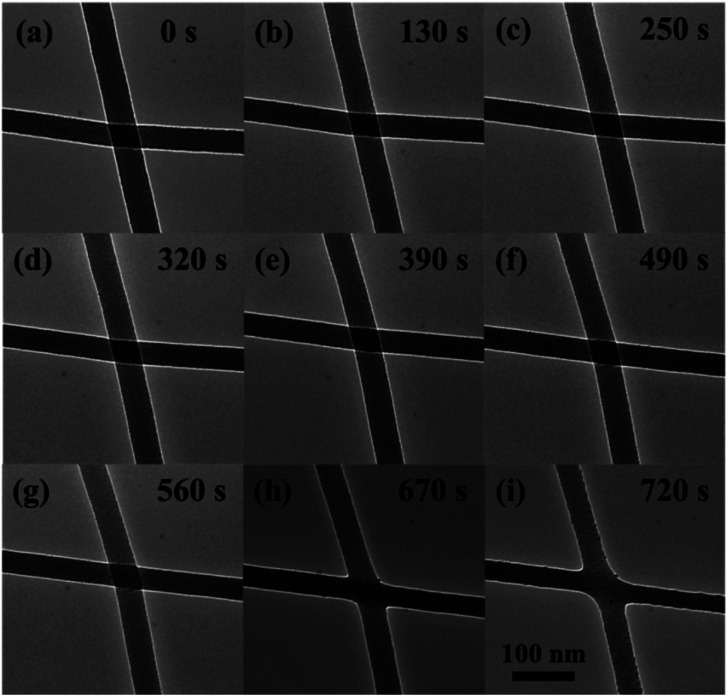
TEM images of the NWs during the welding are shown in (a)–(i). The images show the overall change in the irradiation and the corresponding irradiation time is shown in the up-right of each figure. The radius of NWs keeps almost invariant during the irradiation with only minor radius shrinkage and elongation, as shown in (g)–(i).

Fig. 1 shows a sequence of the TEM images that illustrate the representative local welding in two crossing a-SiO_*x*_ NWs with almost the same diameter of ∼35 nm under the uniform e-beam irradiation. Fig. 1a shows the very initial of two straight and smooth NWs, which cross each other before the irradiation. Fig. 1b–i show a gradual change in the shape, structure, and size of the NWs at the crossing position after the irradiation for different irradiation times (or electron doses). The overall process demonstrated that in about 12 min of the irradiation, the wire segments were demonstrated to particularly maintain their cylindrical shape.

**Fig. 2:**
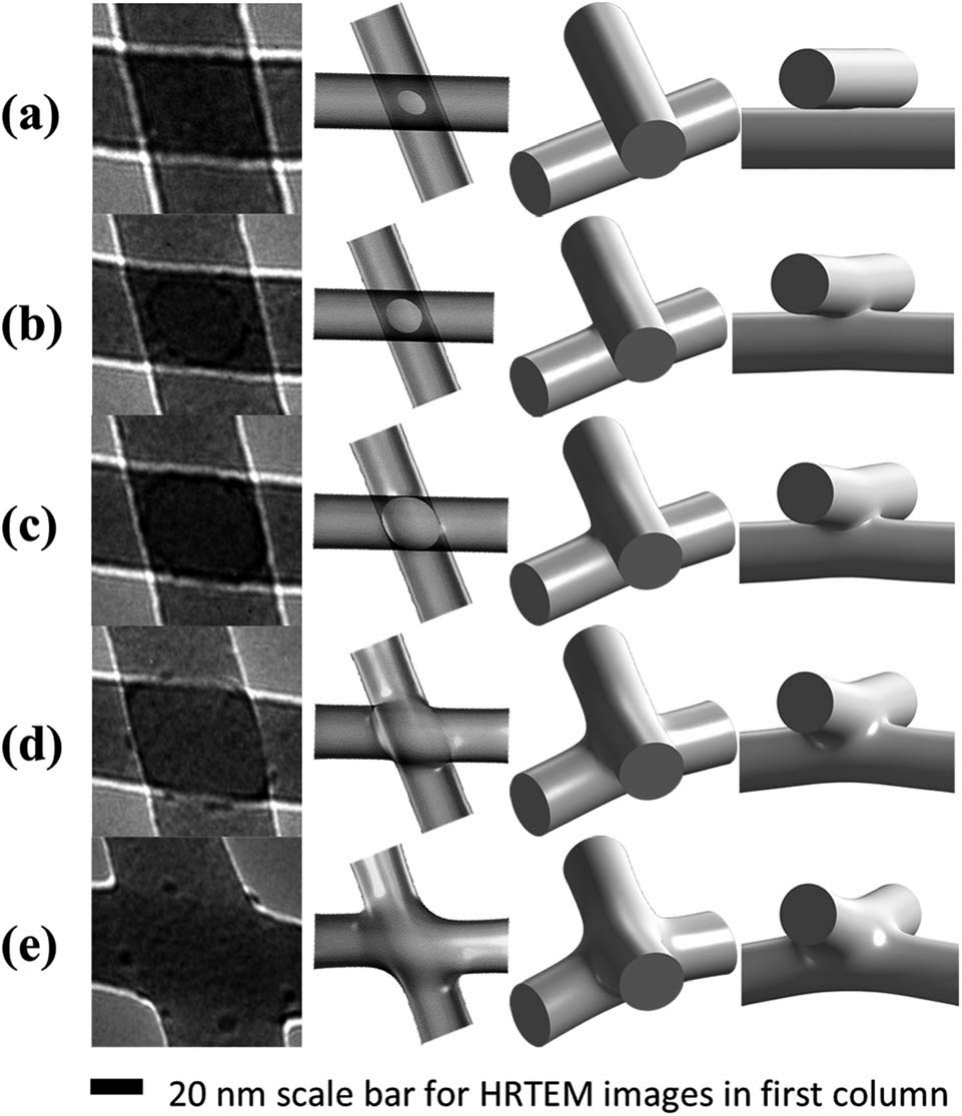
Comparison of the HRTEM images with the simulation results. The welding boundary can be seen in the HRTEM images with high contrast. The simulation result shows more detail in the morphology. The bending of NWs in *z*-axial in simulation is due to the limitation of the size of simulation and is not shown in the experiment.

As the technical limitation of the TEM observation, the image can only reproduce the cross-sectional structure of the LDNs. However, the 3-dimensional structure of the junction is critical for the application. We herein proposed a computation model to reproduce the 3-dimensional structure from the TEM image. In the experiment, the atom diffusion induced a nanostructure change of the NWs *via* plastic flow with the volume of the junction almost invariant. In this way, we propose that this process can be simulated with a combination of existing experimental data and the inherent property of the plastic flow as driven by nanocurvature and athermal activation. The plastic flow observed in the experiment could be described with the Stokes equation of an incompressible flow. In the model, the plastic flow is described by the length scale of the system *L*, surface energy coefficient *σ*, and viscosity *η*. The above three parameters decided time *t* for reaching a given morphology. For such a case, we have a dimensionless number: *c* = *ηL*/*tσ*, which implies the similitude of the model. That is, for models with different parameters (*L*, *σ*, *η*) and a given geometry, if only there exists a transformation between each set of parameters, the geometry evolution and velocity field can be connected with the transformation, and the transformation coefficient is determined by the dimensionless number *c*. In this way, the only uncertainty is the geometry dependence of *η*/*σ*. The determination of this parameter with experimental data is cumbersome and is shown in Appendix. The model was evaluated with COMSOL multiphysics, and the details of the simulation are shown in Appendix. Through the above modeling, we could further study the nanostructure change and the atom diffusion along with the irradiation by numerical calculation. The nanostructure at some stages in Fig. 1 is calculated and shown in Fig. 2. In Fig. 2, the calculation result is compared with the high-resolution TEM (HRTEM) micrographs of the representative junctions before and after irradiation, as the same shown in Fig. 1a, c, e, g and i. As shown in Fig. 2, during an initial stage of the e-beam irradiation, there is a distinct welding boundary formed at the junction (as indicated in Fig. 1c and 2b). With the increase in the irradiation time, the welding boundary extended outward significantly (as indicated in Fig. 1d–f and 2b–d), and then, the welding boundary on the junction regions became vaguer and vaguer (as indicated in Fig. 1g and 2d). As further extending the irradiation time, the crossed position became smooth (as shown in Fig. 1g–i, 2d and e). Moreover, Fig. 1g demonstrates a minor preferential locally radial shrinkage from the NWs nearby the junction and an increase in the welded junction area, which were also observed throughout the remaining irradiation. Finally, the welding boundary disappeared (as shown in Fig. 1h and 2e). We can see from the 3-dimensional structure shown in Fig. 2 that in the stage from Fig. 2a–c, there exists an area around the welding boundary with a high negative curvature. This area is not only attractive to migrate atoms but also preferentially breaks under an external force. A similar irradiation on different crossing wire segments was repeated several times. We observed that the features of the structural changes are essentially the same, as shown in Fig. 1 and 2.

Evidently, such a fast welding and x-type deformation was attributed to the novel, athermally activated, collective diffusion or plastic flow of a large number of atoms rather than the atomic loss as predicted by the existing knock-on mechanism.^14,15,20,21,23^ Therefore, we predict that these can be well interpreted by the diffusion mechanism of surface atoms, which is proposed on the basis of the nanocurvature effect^26,27^ and the e-beam-induced athermal activation effect.^26,28^ For the nanocurvature effect on a NW, we can suppose that similar to the nanoparticle case as described in our previous studies,^24,27^ when the diameter of a NW approaches its atomic bond length, a positive nanocurvature on the radially-curved wire surface will become appreciable. Such a positive nanocurvature would cause an additional tensile stress on the electron cloud structure of the surface atoms, which would lead to a dramatic increase in the surface energy. This dramatically increased surface energy would lower the energy barrier for the migration of atoms, resulting in a tendency for a NW to shrink (Fig. 1). It also has been observed that the ‘Debye temperature’ or melting point^23^ of the LDN is lower than that of the bulk material^33^ because of the surface positive nanocurvature and the low dimension of the LDN. It can be asserted that a similar tendency would also occur in a NW. Thus, it can be further inferred that with the near-surface tensile stress, the thermal vibration frequencies of near surface atoms in a NW would decrease, and thus the ‘Debye temperature’ would be lower, and cause the wire sidewall to melt, and the atoms therein to migrate or escape out. However, for the two crossing NWs, as shown in Fig. 1a, besides the radial positive nanocurvature, there is a negative nanocurvature further produced on the surrounding of the contacting point. The negative nanocurvature would cause an additional compressive stress on the electron cloud structure of surface atoms in the surrounding of the contacting point. This additional compressive stress may prompt dramatic increase in the surface energy, which can cause a strong structural change for two crossing NWs to weld (see Fig. 1). In other words, the compressive stress would possibly lead to a speeding up of the vibration of the surface atoms, and thus increase the ‘Debye temperature’, and induce the crossing NWs of nearby contacting position to get other atoms. That is, the ‘Debye temperature’ of the negative curvature location (or contacting position) is lower than that of the positive curvature location (or wire sidewall), the wires sidewall are ‘hotter’ than the contacting position if the wires contact with each other. In this way, the positive or negative nanocurvature would cause intrinsic structural instabilities for the two crossing NWs. The result is that the surface atoms near the junction position are adsorbed by the contacting point, thus further facilitating the welding at the NW junction.

For the two crossing NWs, although the nanocurvature (including the positive and negative nanocurvature) with the low dimension can suppress the energy barrier for atoms to diffuse (or migrate), thus causing structural instability, further assistance from an external excitation such as energetic beam irradiation is still needed to finally realize the ultrafast mass transportation and structure changes. Our *in situ* observations in the present work demonstrate that when the beam energy deposition rate of an incident energetic beam approaches the atomic vibration frequency, the atoms would not have enough time to convert the deposited beam energy to their thermal vibration energy *via* most of the inelastic interaction processes. Thus, the mode of atomic thermal vibrations would become very soft or the atomic vibrations would lose stability, which can further suppress the energy barrier for the atoms to migrate, and finally make the structure changes kinetically possible. Note that the beam energy deposition rate on the specimen was used in this article as a key parameter to account for the irradiation-induced athermal activation processes. When the beam energy deposition rate is relatively low, the soft mode and instability of atomic vibration would lower the energy barrier between atoms for their diffusion, and induce a high mobility of the atoms and thus the athermal plastic flow, migration or diffusion of the atoms would predominate (see Fig. 1 and 2). This phenomenon was called the beam-induced athermal activation effect (or nanotime effect).^25,26^

**Fig. 3:**
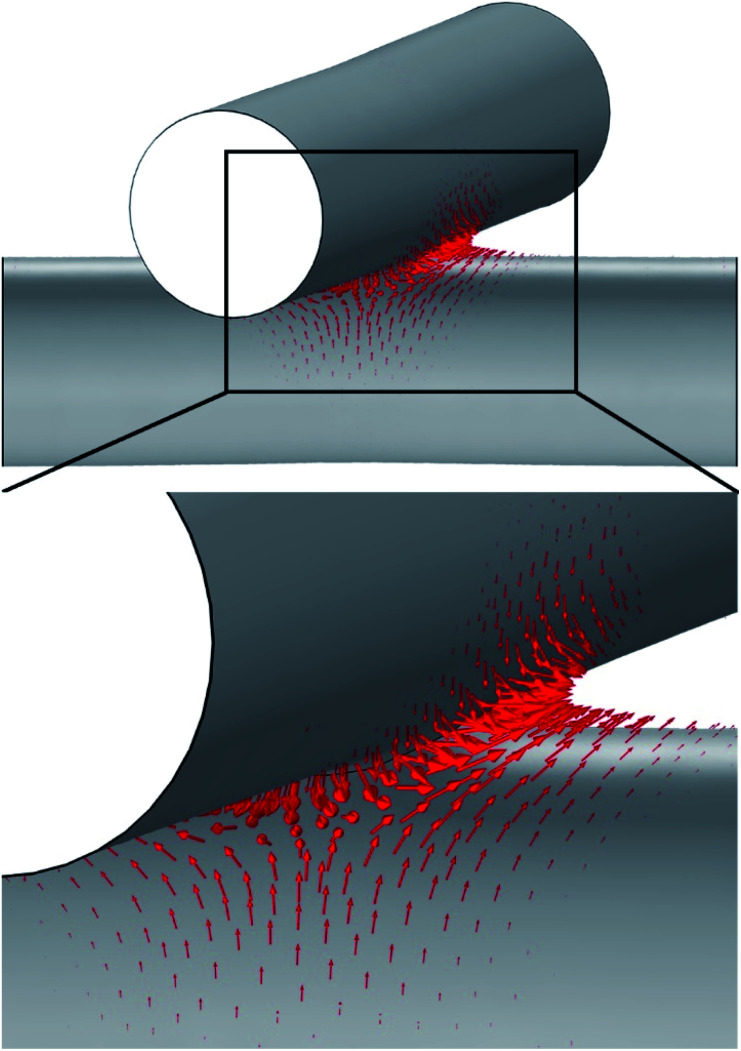
Diffusion field on the surface of the NWs at the early stage. The length of arrows is in proportion to the logarithm of the diffusion velocity to base 150. The arrows show that the diffusion in the early stage is very strong in boundary with negative curvature compared to the surface with positive one. The direction of arrows show that the diffusion tendency is from the positive curvature point to the negative curvature point.

In order to further study the nanowelding process of the two crossing NWs, the diffusion velocity field was calculated, as shown in Fig. 3. The tangential component of the arrows indicates the speed and direction of migrate atoms, and the normal component indicates the condensation speed of migrate atoms. As shown in the figure, the diffusion velocity shows very peaky distribution on the surface. The diffusion on the surface with a negative curvature with a larger difference of nanocurvature with its surrounding surface is significantly stronger than the other region. Moreover, the direction of the velocity field showed that the diffusion is from the sides with higher (more ‘positive’) curvature towards sides with a lower (more ‘negative’) curvature. As the consequence of the diffusion velocity field, the diffused atoms will accumulate in the position with negative curvature in an unexpected speed; in this way, the diffusion would gradually slow down. In summary, under the athermal activation of the e-beam irradiation, the surface atoms near the contacting point of the two crossing NWs were preferentially desorbed (or migrated) to the contacting point. That is, the surface atoms would tend to diffuse preferentially from one position with the positive nanocurvature to another with the negative nanocurvature. In order to reduce the surface energy of the system, the NWs showed a preferential locally radial shrinkage through the diffusion of near-surface atoms to the junction position. Consequently, increasing number of atoms gathered in the junction position, causing two crossing NWs to be welded locally in the junction regions, and there was a significant weld boundary appearing. Moreover, the surface atoms of the junction regions preferentially gathered up in the direction perpendicular to the axis with a larger curvature, leading to the formation of the welding boundary of an irregular elliptical shape (*cf.*Fig. 2b and c). Eventually, the crossed position became round to minimize the total energy of the two welded NWs. Clearly, such an accelerating necking and x-type deformation is believed to be caused when the quick axial collective diffusion to the four ends of the welded wires was somewhat hindered by the four supporting edges of the carbon film holes. Importantly, we observed that even after the above accelerating local radial shrinkage with the fast diffusion and the x-type deformation processed for a time, it will stop immediately once the irradiation is suspended. This means that the process is predominately driven by ultrafast (or instant) irradiation-induced athermal activation, but not by a slow (or retarded) beam heat-induced thermal activation. Therefore, we predicted that there must be two mechanisms, which are intrinsic to the crossed amorphous NWs under the irradiation to drive the athermal plastic flow of massive atoms: (1) the high surface energy of the NW gives rise to a strong, thermodynamic tendency for the wires to self-contract, and (2) the e-beam irradiation athermally softens the welded wires and kinetically activates the contraction and the plastic flow. Irradiation-induced fast diffusion of atoms has also been observed to induce straightening of bent SiO_*x*_ nanowires^34^ and elongation together with the radial shrinkage of straight SiO_*x*_ nanowires.^35^ Moreover, gold nanoparticles^36^ have been observed to coalesce under uniform e-beam irradiation. These phenomena are driven by the structural instability with the help of the athermal activation effect of e-beam irradiation.

The current density or irradiation dose rate is crucial to the athermal welding technique. The current density applied in the present welding was 1 A cm^−2^. Under such current density, the beam-induced phenomenon was dominated by the plastic flow and the diffusion of atoms. However, for a much higher current density,^21^ the beam-induced phenomenon will be mainly the evaporation of atoms. This evaporation of atoms shows preference on the surface with high positive curvature. It could also be utilized for flexible welding of SiO_*x*_ nanowires to carbon films.^37^

## Conclusions

In summary, the effects of nanocurvature and e-beem induced athermal activation on local nanowelding of two crossing a-SiO_*x*_ NWs were *in situ* investigated in TEM. It was observed that at room temperature, the two crossing NWs demonstrated a local welding and an accelerated local radial shrinkage at the nanoscale. In addition, the preferential local diffusion and plastic flow of wire surface atoms from near the contacting point of the wire sidewalls to the contacting point demonstrated the effect of positive and negative nanocurvatures non-uniformly distributed in the two crossing NWs. The above processes involve accelerated, athermal migration of surface atoms and could not be adequately explained by the existing knock-on mechanism and electron beam heating effect. However, it can be well interpreted by a novel mechanism of the athermal diffusion and plastic flow of wire atoms as driven by the nanocurvature of a-SiO_*x*_ NWs and the e-beam-induced soft mode and instability of atom vibration. The study has also important implications for the fabrication, processing and stability of future NW-based structures or devices. Thus, the study is crucial not only to the fundamental understanding, but also to the technical controlling over the e-beam-induced nanoinstability and nanoprocessing of LDNs.

## Appendix

The simulation is conducted by solving the Stokes equation of incompressible flow:

A1 ∇·

<svg xmlns="http://www.w3.org/2000/svg" version="1.0" width="17.272727pt" height="16.000000pt" viewBox="0 0 17.272727 16.000000" preserveAspectRatio="xMidYMid meet"><metadata>
Created by potrace 1.16, written by Peter Selinger 2001-2019
</metadata><g transform="translate(1.000000,15.000000) scale(0.015909,-0.015909)" fill="currentColor" stroke="none"><path d="M160 840 l0 -40 40 0 40 0 0 -360 0 -360 -40 0 -40 0 0 -40 0 -40 200 0 200 0 0 40 0 40 -40 0 -40 0 0 160 0 160 160 0 160 0 0 40 0 40 40 0 40 0 0 160 0 160 -40 0 -40 0 0 40 0 40 -320 0 -320 0 0 -40z m240 -400 l0 -360 -40 0 -40 0 0 360 0 360 40 0 40 0 0 -360z m240 200 l0 -160 -80 0 -80 0 0 160 0 160 80 0 80 0 0 -160z m160 0 l0 -160 -40 0 -40 0 0 160 0 160 40 0 40 0 0 -160z"/></g></svg>

 = 0

A2 = *pI* + *η*[∇*

<svg xmlns="http://www.w3.org/2000/svg" version="1.0" width="13.454545pt" height="16.000000pt" viewBox="0 0 13.454545 16.000000" preserveAspectRatio="xMidYMid meet"><metadata>
Created by potrace 1.16, written by Peter Selinger 2001-2019
</metadata><g transform="translate(1.000000,15.000000) scale(0.015909,-0.015909)" fill="currentColor" stroke="none"><path d="M480 840 l0 -40 -160 0 -160 0 0 -40 0 -40 160 0 160 0 0 -40 0 -40 40 0 40 0 0 40 0 40 40 0 40 0 0 40 0 40 -40 0 -40 0 0 40 0 40 -40 0 -40 0 0 -40z M80 520 l0 -40 40 0 40 0 0 -40 0 -40 40 0 40 0 0 -200 0 -200 40 0 40 0 0 40 0 40 40 0 40 0 0 40 0 40 40 0 40 0 0 40 0 40 40 0 40 0 0 40 0 40 40 0 40 0 0 120 0 120 -80 0 -80 0 0 -40 0 -40 40 0 40 0 0 -80 0 -80 -40 0 -40 0 0 -40 0 -40 -40 0 -40 0 0 -40 0 -40 -40 0 -40 0 0 160 0 160 -40 0 -40 0 0 40 0 40 -80 0 -80 0 0 -40z"/></g></svg>

* + (∇**)^*T*^]

A3 ∇·** = 0

In the model, the athermal activation effect is modeled in the viscosity which depends on the irradiation dose rate and the nanocurvature effect is modeled in the free energy which depends on the surface nanocurvature or nanowire radius. The nanocurvature effect manifests in the boundary condition:

A4 *n⃑*·ℙ = *σ*(∇_*t*_·*n⃑*)*n⃑*

The above model gives a velocity field from a given geometry and parameters (*η*,*σ*) where the geometry evolves accordingly.

**Fig. 4:**
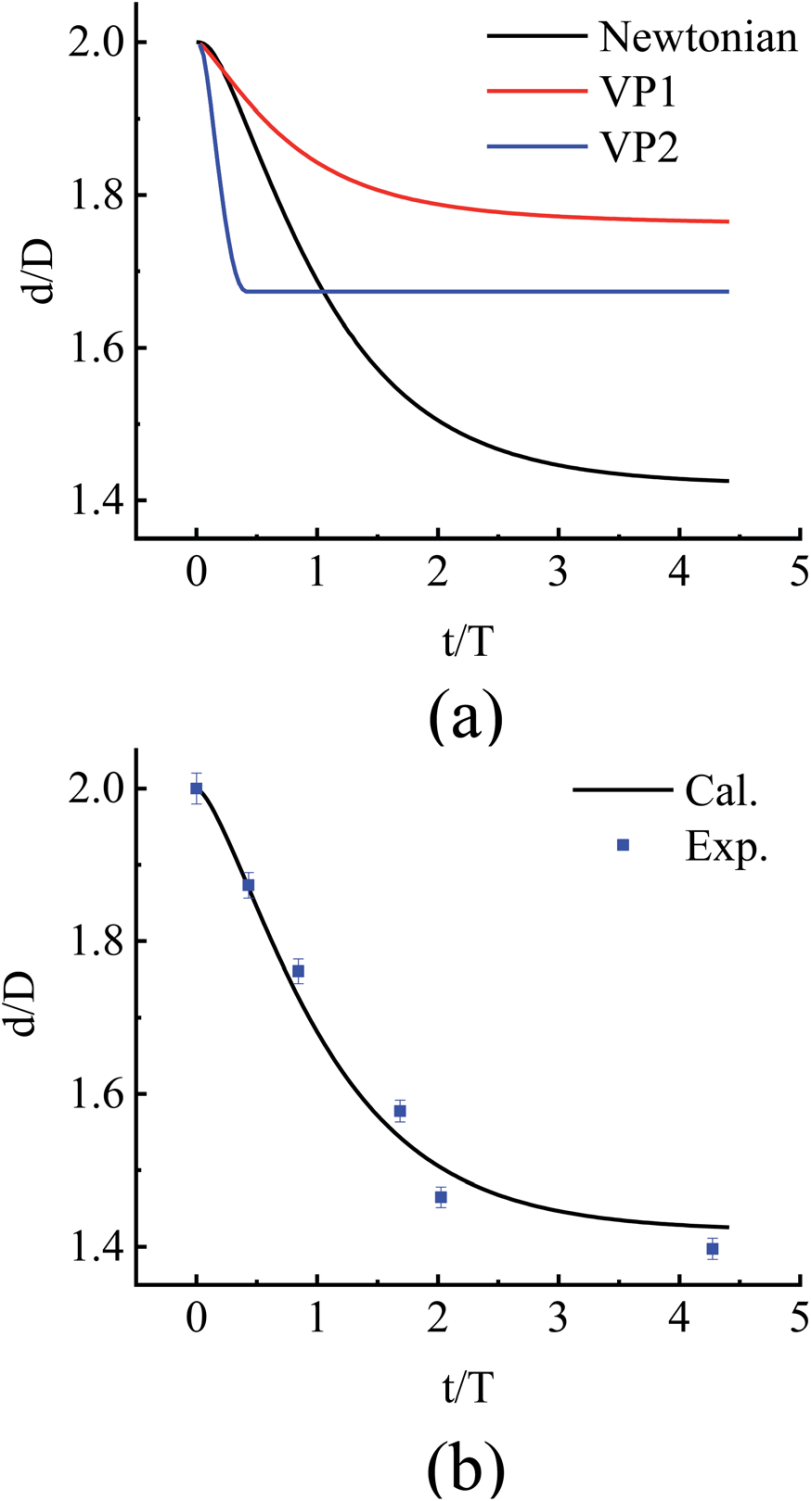
The evolution of *d*/*D* with respect to irradiation time *t* (or dose). The data are nondimensionalized with the diameter of nanowire (*D*) and the irradiation time (*T*) when the distance *d* between the two axes of the parallel and contacted amorphous SiO_*x*_ nanowires reaches 
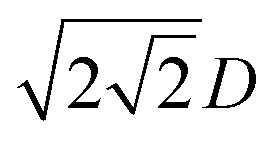
. (a) Comparison for the three different models with different type of *η* where the welding would not complete perfectly with a considerable increase of *η* when surface curvature decreases to a certain value. (b) Fitting to the experiment data. The fitting curve shows some minor errors due to the evaporation of atoms in the irradiation. The fitting agrees well with the Newtonian model where the viscosity *η* is almost invariant.

A set of the measured data from paralleled coalescence of SiO_*x*_ nanowires with the similar radius and irradiation dose rate^33^ is used to determine how the parameter *η*/*σ* varies according to the geometry. The result is shown in Fig. 4. Three different models were taken into account for comparison, where the Newtonian model represent the case where *η*/*σ* is invariant in the process, the VP1 model and the VP2 model correspond to the cases where *η*/*σ* increase along the decrease of surface curvature with different arguments. Compared to the model where *η*/*σ* increase with the decrease of surface curvature, the experiment data shows that *η* is almost constant in the coalescence. The uniform irradiation configuration induces a uniform athermal activation effect.

With the parameter determined, the model is then applied to the cross welding configuration and calculated with finite element method.

## Conflicts of interest

There are no conflicts to declare.

## Supplementary Material

RA-012-D1RA08176D-s001
