# Supplementary material for: Electron beam-induced athermal nanowelding of crossing SiOx amorphous nanowires
Source: RSC Adv. 2022 Feb 21;12(10):6018–24. doi: 10.1039/d1ra08176d (PMC8981572; doi:10.1039/d1ra08176d)
Supplement: RA-012-D1RA08176D-s001 [file RA-012-D1RA08176D-s001.pdf]

Electronic Supplementary Material (ESI) for RSC Advances.
This journal is © The Royal Society of Chemistry 2022

Appendix

The simulation is conducted by solving the Stokes equation of incompressible flow:

$$\nabla \cdot p = 0\#(A1)$$

$$p = -pI + \eta\left[\nabla \vec{v} + (\nabla \vec{v})^T\right]\#(A2)$$

$$\nabla \cdot \vec{v} = 0\#(A3)$$

In the model, the athermal activation effect is modeled as the viscosity, and the nanocurvature effect is modeled as the free energy depends the surface area. The nanocurvature effect manifests in the boundary condition:

$$\vec{n} \cdot p = \sigma\left(\nabla_t \cdot \vec{n}\right)\vec{n}\#(A4)$$

The above model gives a velocity field from a given geometry and parameters (η,σ), and geometry evolves accordingly.

Measured data from paralleled coalescence of SiOx nanowires with approximative radius and irradiation dose rate[1] is used to determine how the parameter η/σ vary according to geometry. The result is shown in Fig.A1. Three different models were taken into comparison, where Newtonian represent the case where η/σ is invariant in the process, VP1 and VP2 correspond to the cases where η/σ increase along the increase of surface curvature with different arguments. Compared to the model where η/σ increase respect to increase of negative surface curvature, experiment data shows that η/σ is almost constant in the coalescence. The uniform irradiation configuration induces a uniform athermal activation effect.

With the parameter clear, the model is then applied to cross welding configuration

and calculated with finite element method.

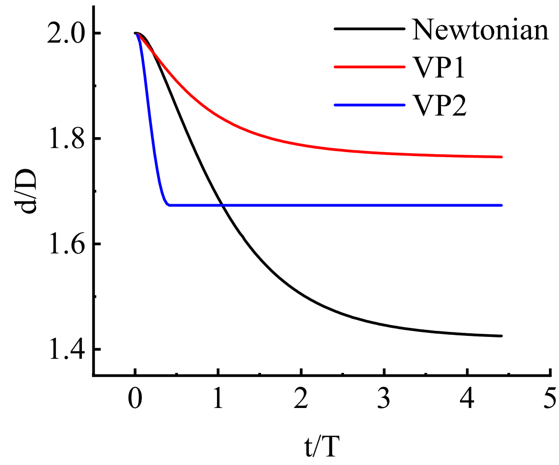

(a)

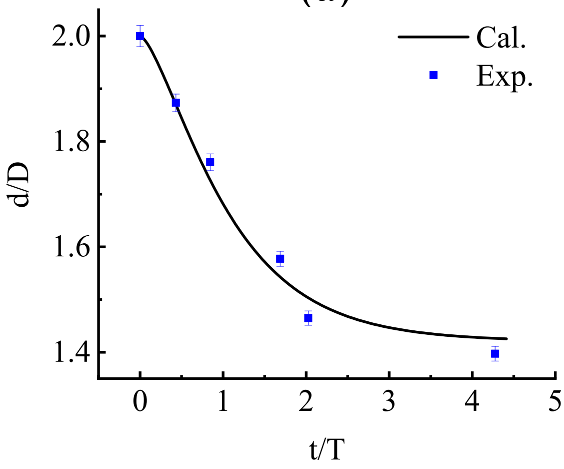

(b)

Fig.A1 The evolution of total wide of two parallel contact amorphous SiOx nanowires respect to irradiation time(or dose). The data are nondimensionalized with D being the diameter of nanowire and T being the time that the total wide reaching $d=\sqrt{2\sqrt{2}}D$. (a) Comparison between different the variable dependence of η/σ, the welding would not complete perfectly with a considerable increase of η/σ when surface curvature increases to a certain value. (b) Fitting to experiment data. The fitting curvature shows minus error due to evaporation of atoms in irradiation. The viscosity

η/σ is almost invariant.
